# Bacteriome of Moist Smokeless Tobacco Products Consumed in India With Emphasis on the Predictive Functional Potential

**DOI:** 10.3389/fmicb.2021.784841

**Published:** 2021-12-24

**Authors:** Mohammad Sajid, Sonal Srivastava, Amit Kumar, Anuj Kumar, Harpreet Singh, Mausumi Bharadwaj

**Affiliations:** ^1^Division of Molecular Genetics and Biochemistry, Molecular Biology Group, ICMR-National Institute of Cancer Prevention and Research, Noida, India; ^2^ICMR-AIIMS Computational Genomics Centre, Division of Biomedical Informatics, Indian Council of Medical Research (ICMR), New Delhi, India; ^3^Molecular Biology Group, ICMR-National Institute of Cancer Prevention and Research, Noida, India

**Keywords:** smokeless tobacco products (STPs), tobacco-specific nitrosamines (TSNAs), nitrogen metabolism genes, smokeless tobacco-associated bacteriome, antibiotic-resistance genes, toxins, oral cancer

## Abstract

Smokeless tobacco products (STPs) carry assorted microbial population that contributes to carcinogens synthesis like tobacco-specific nitrosamines (TSNAs). Extensive exploration of microbiota-harboring STPs is required to understand their full carcinogenic potential. Here, we applied 16S rRNA gene sequencing to investigate bacteriome present in moist STPs immensely consumed in India (*Khaini, Moist-snuff, Qiwam*, and *Snus*). Further, the functional metagenome was speculated by PICRUSt (Phylogenetic Investigation of Communities by Reconstruction of Unobserved States) to assign the abundance of genes related to nitrogen metabolism, bacterial toxins, antibiotic drug resistance and other pro-inflammatory molecules. Highly diverse bacterial communities were observed in all moist STPs. Taxonomic analysis revealed a total of 549 genera belonging to four major phyla *Proteobacteria*, *Firmicutes*, *Bacteroidetes* and *Actinobacteria*. Overall, the core bacterial genera *Acinetobacter*, *Bacillus*, *Prevotella*, *Acetobacter*, *Lactobacillus*, *Paracoccus*, *Flavobacterium*, and *Bacteroides* were significantly abundant in moist STPs. Elevated moisture-holding products like *Moist-snuff* and *Qiwam* harbor rich bacterial species diversity and showed similar bacteriome composition. Furthermore, *Qiwam* products showed the highest level of genes associated with nitrogen metabolism, antibiotic resistance, toxins, and pro-inflammation (predicted by PICRUSt) which can contribute to the synthesis of TSNAs and induction of oral cancer. The present broad investigation of moist STPs-associated bacteriome prevalence and their detailed metabolic potential will provide novel insight into the oral carcinogenesis induced by STPs.

## Introduction

Non-combustible form of tobacco (smokeless tobacco) is used by >300 million people worldwide and majorly (>85%) in South Asian countries (Siddiqi et al., 2020). A report on global tobacco consumption documented that there are 199.4 million smokeless tobacco (SLT) users in India (GATS-2, 2017). SLT consumption is associated with cancer, cardiovascular diseases, nicotine addiction, diabetes mellitus, oral diseases, neuronal and reproductive defects (Carlsson et al., 2017; Centers for Disease Control and Prevention, 2020). These pathological effects are generally assigned to carcinogens present in STPs essentially tobacco-specific nitrosamine (TSNAs), benzo(a)pyrene and heavy metals (Critchley and Unal, 2003; Stepanov et al., 2008). Among 233 unique chemical compounds reported in STPs, TSNAs are the most abundant and potent carcinogens due to their high toxicity and ability to reprogram normal cells into neoplastic cells (Kaur et al., 2019; Sarlak et al., 2020). The STPs consumed in the South Asian region have higher TSNAs content in contrast to STPs available in Western countries (Nasrin et al., 2020).

The moist STPs are the most commonly used non-combustible variants of tobacco in India (GATS-2, 2017). The various categories of moist SLT products available in India include *Khaini*, *Moist-snuff*, *Qiwam* (*Kiwam*), and *Snus* (Gupta and Ray, 2003). The SLTChemDB database provides details about the physicochemical properties, biological information, toxicological effects and information of chemicals found in moist STPs (Kaur et al., 2019). The moist STPs are not only different in their composition and manufacturing process but also possess variable levels of TSNAs (Stepanov et al., 2017; Kaur et al., 2019; Shaik and Maddu, 2019). Moisture content and storage temperature are significantly responsible for the synthesis of high levels of TSNAs in tobacco products (Shi et al., 2013; Law et al., 2016). Storage conditions and aging also influence the TSNA levels in STPs that may be due to changing microbial diversity within these products (Chong et al., 2020).

The microbiota has been previously reported in cured tobacco leaves and STPs using both culture-dependent and culture-independent approaches (Di Giacomo et al., 2007; Han et al., 2016; Tyx et al., 2016; Smyth et al., 2017; Mehra et al., 2020). The microbiome of tobacco products participates in the synthesis of TSNAs. These microbes produce nitrite from nitrate and these nitrite molecules further react with various alkaloids available in the tobacco products and generate TSNAs (Shi et al., 2013; Chopyk et al., 2017). Studies on the SLT associated bacteriome showed the presence of species belonging to clinically relevant bacterial classes like Actinobacteria (*Corynebacterium, Mycobacterium*, and *Propionibacterium*), Bacteroidia (*Prevotella* and *Porphyromonas*), Bacilli (*Bacillus, Listeria, Staphylococcus*, and *Streptococcus*), Chlamydiae, Clostridia (*Clostridium*), Fusobacteria (*Fusobacterium* and *Streptobacillus*), α-Proteobacteria (*Brucella* and *Rickettsia*), β-Proteobacteria (*Neisseria* and *Spririllum*), γ-Proteobacteria (*Haemophilus* and *Pseudomonas*), Spirochete (*Borrelia* and *Leptospira*) and Mollicutes (*Mycoplasma*) (Gholizadeh et al., 2016; Al-Hebshi et al., 2017; Monika et al., 2020). Furthermore, SLT-associated microbes can influence systemic inflammation and supplementary signaling pathways associated with oral cancer progression because they produce toxins and other pro-inflammatory molecules (Rivera et al., 2020; Sajid et al., 2021). Additionally, SLT-associated microbiota can be contaminated with pathogenic microbes and possibly a source of antibiotic resistance threat (Rivera et al., 2020).

Despite the significant potential of STP associated microbes to form TSNAs, their presence in various STPs is still not well explored and significant metagenome analysis has not been conducted on STPs, especially those available in India. Therefore in this study, we have selected 11 moist SLT products based on high consumption and elevated moisture content which can facilitate microbial growth inside these STPs. The bacterial community structure was investigated by 16S rDNA and prediction of critical functional genes was performed related to nitrogen metabolism, antibiotic drug resistance, production of toxins and pro-inflammatory molecules. Hence, comprehensive metagenome analysis was performed using the MicrobiomeAnalyst platform to unveil the structural and functional bacteriome present in moist STPs especially consume in India.

## Materials and Methods

### Tobacco Samples and DNA Extraction

The domestic moist STPs were purchased from vendors in the Delhi and Uttar Pradesh state of India. The 16S rDNA sequencing techniques were used to analyze the bacterial diversity of 11 moist STPs from 4 different categories including *Khaini* (K1, K2, and K3), *Moist-snuff* (MS1, MS2, and MS3), *Qiwam* (Q1, Q2, and Q3), and *Snus* (S1 and S2) samples. The STPs were stored at −20°C to inhibit the further growth of microorganisms. The STPs were open in the sterilized conditions and microbial metagenomic DNA was isolated with a Power-soil DNA isolation kit as per the protocol provided by the manufacturer (Qiagen, Bangalore, India). The purity and quantity of isolated metagenomic DNA from the different STPs was checked by NanoDrop (Thermo, Bangalore, India). Further, the quality of metagenomic DNA was confirmed agarose gel (1%) before amplification by PCR. The metagenomic DNA concentration of all tested SLT products was found to be >30 ng/μl.

### Bacterial 16S rDNA Amplification and Library Preparation

The PCR was performed to amplify the 16S rDNA V_3_–V_4_ region of metagenomic DNA isolated from the different moist STPs. The extracted metagenomic DNA (40 ng) was amplified with a pair of universal primers (10 pM of each) (FP: 5′-AGAGTTTGATGMTGGCTCAG-3′ and RP: 5′-TTACCGCGGCMGCSGGCAC-3′) as described earlier (Kroes et al., 1999). Along with primers and metagenomic DNA, a master mix was added containing dNTPs (0.5 mM), MgCl_2_ (3.2 mM), high-fidelity DNA polymerase and PCR enzyme buffer. The PCR amplification was performed with the following condition: 95°C for 3 min chase by 25 cycles at 95°C/15 s, 60°C/15 s and 72°C for 120 s and with a final elongation at 72°C for 10 min. The amplified 16S PCR amplicons were purified and subjected to agarose gel (2%) and NanoDrop for quality check.

### Sequencing of Prepared Library

The Ampure beads (Beckman Coulter Inc., Indianapolis, IN, United States) were used to purify the amplicons of each sample by eliminating the unused primers and to prepare the sequencing libraries an additional 8 cycles of PCR was executed via Illumina barcoded adapters (Supplementary Table 1). Further, prepared libraries were purified by Ampure beads and quantified with the help of a QuDye-dsDNA HS assay kit (Thermo Fisher, Bangalore, India). The Illumina Miseq with a 2 × 300PE v3 sequencing kit was used for sequencing at Biokart India Pvt. Ltd., Bengaluru, India. The raw data was submitted to NCBI Short Read Archive (SRA) under BioProject accession number PRJNA767533.

### Bioinformatics Analysis

The raw data binary base call (BCL) file received from the sequencer was de-multiplexed into FASTQ format. Quality control checks of raw sequenced data were performed by FastQC (Version 0.11.9) and MultiQC (Version 1.10.1) tools. Next, removal of contaminant adapters and trimming of low-quality reads were done by TrimGalore (Version 0.6.6)^1^. The QC passed samples were again analyzed by Quantitative Insights Into Microbial Ecology (QIIME version 1.9.0) workflow including fusion of paired-end reads, chimera elimination, OTU clustering and taxonomy assignment (Caporaso et al., 2010). The QIIME workflow facilitates precise exploration at the genus level. The Kraken2 with database NCBI was used as a reference for OTU picking (Wood et al., 2019). Further, data was filtered on a web-based platform MicrobiomeAnalyst to eliminate the low quality or uninformative features using minimum count = 4, 20% prevalence and low variance filter (Inter-Quartile range – 10%) (Chong et al., 2020). A total of 148 low abundance features were separated based on prevalence and a total of 28 low variance features were removed based on inter-quartile range. The 247 number features remain after the data filtering step. All analyses like rarefaction curve, α-diversity, principal coordinate analysis (PCoA), core microbiome, cluster study, random forest, Linear discriminant analysis Effect Size (LEfSe) and Sparse Correlations for Compositional data (SparCC) were performed by MicrobiomeAnalyst^2^. Subsequently, the metabolic pathway analysis was executed using the PICRUSt (Phylogenetic Investigation of Communities by Reconstruction of Unobserved States) algorithm (Langille et al., 2013). The functional genes associated with bacteriome of moist STPs were derived from the Kyoto Encyclopedia of Genes and Genomes (KEGG) database (Kanehisa and Goto, 2000). Next, Taxon Set Enrichment Analysis (TSEA) module of MicrobiomeAnalyst was performed to identify the biologically or ecologically meaningful patterns of STP associated bacteriome by analyzing them with context to pre-defined taxon set. To check the reproducibility of sequencing results, duplicates of *Snus* samples (S1_dup and S2_dup) were taken.

### Statistical Analysis

The relative abundance percentage of each OTUs of taxonomic classification was calculated and plotted using Origin software. For data set phyla, we removed OTUs < 10 reads and for the data set genus, we removed OTUs < 100 reads. Analysis of Variance (ANOVA) was used to determine differences between products. A *p*-value < 0.05 was considered statistically considerable.

## Results

### Bacterial Community Composition and Diversity in Moist Smokeless Tobacco Products

After all pre-processing steps, an OTU table was generated and the number of amplicons obtained for each moist STPs varies between 10,601 and 131,082 (Supplementary Table 2 and Supplementary Figure 1). Most of the moist STPs showed an increase in observed bacterial species richness and diversity (Supplementary Figure 2). The numbers of OTUs were found to increase as the sequencing depth increases and saturation of species richness was observed in the rarefaction curves for all the tested moist STPs (Supplementary Figure 2). However, the numbers of observed bacterial species were less in K3 and S2 as compared to other moist STPs. Interestingly, the STPs having the highest moisture content such as *Moist-snuff* and *Qiwam* exhibited higher overall species diversity than *Khaini* and *Snus* which have comparatively low moisture content (Supplementary Figure 2 and Supplementary Table 3).

Further, OTUs richness was estimated by determining α-diversity (within-sample diversity) indices (Chao1, Fisher, Shannon, and Simpson) of STP-associated bacteriome. The *Moist-snuff* and *Qiwan* showed increased diversity indices as compared to *Khaini* and *Snus* suggesting that the *Moist-snuff* and *Qiwam* products have higher expected species richness of the bacteriome (Supplementary Figure 3 and Table 1). However, there was no statistically significant change observed among the four groups of moist STPs (Supplementary Figure 3). All the moist STPs displayed Good’s estimator values > 99% suggesting that the majority of bacterial species in the sample have been detected (Table 1).

**TABLE 1:** Diversity indices and estimator among moist smokeless tobacco products.

S. no.	Category	Sample ID	Diversity indices and estimator				
			Chao1	Fisher	Shannon	Simpson	Good’s coverage (%)
(1)	*Khaini*	K1	240.50	41.769	3.647168	0.911116	99.80941
		K2	224.03	36.550	3.048932	0.865712	99.68553
		K3	156.50	19.944	0.737206	0.207133	99.80941
(2)	*Moist snuff*	MS1	245.00	40.231	4.035496	0.958174	99.79036
		MS2	244.58	41.769	3.953061	0.953286	99.79036
		MS3	227.00	40.231	4.144013	0.967637	99.88565
(3)	*Qiwam*	Q1	244.50	42.210	4.076863	0.962277	99.78083
		Q2	236.18	41.769	3.849102	0.946535	99.81894
		Q3	235.17	41.328	4.112467	0.963786	99.85706
(4)	*Snus*	S1	234.76	38.707	4.071057	0.965034	99.79036
		S1_dup	228.40	38.707	4.064247	0.965125	99.81894
		S2	199.78	30.039	2.763917	0.843396	99.64742
		S2_dup	195.03	29.834	2.790232	0.844324	99.67601

**FIGURE 1:**
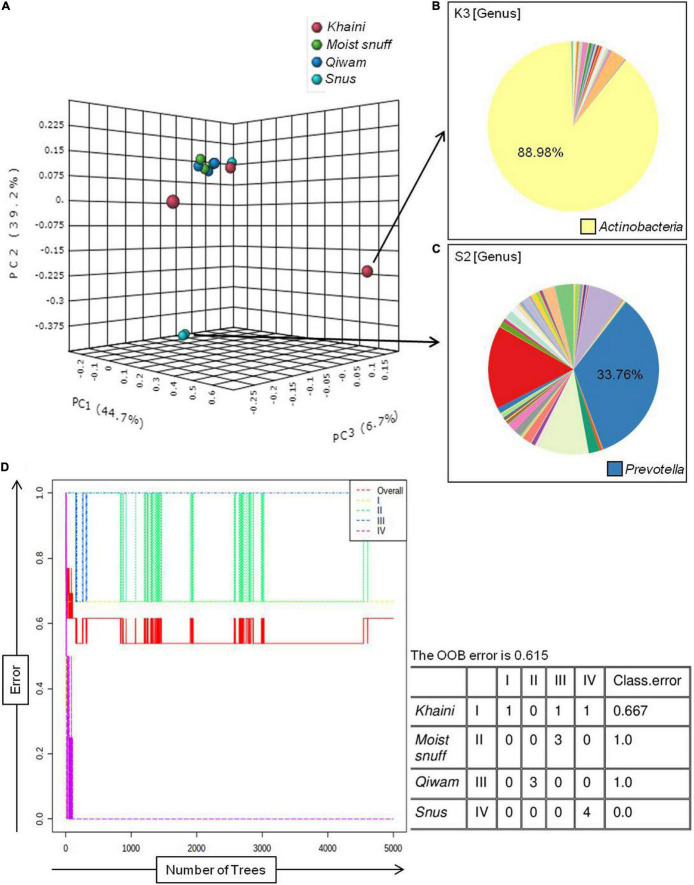
β–diversity among moist smokeless tobacco products. Interactive 3D-Principal Coordinate Analysis (PCoA) plot for bacterial β-diversity in moist STPs and pie chart generated by the MicrobiomeAnalyst. **(A)** PCoA plot of 11 moist STPs derived from Bray–Curtis index showing the distance of bacterial communities present in *Khaini, Moist-snuff, Snus*, and *Qiwam* samples. The samples of each group are represented by different color as indicated on the above of the figure. The pie charts, **(B)** *Khaini* sample, K3 and **(C)** *Snus* sample, S2 are shown at the level of genera. **(D)** The error plot originated from random forest analysis. Overall genera present in moist STPs was represented by a red line, yellow-line indicate the distinct genera present in *Khaini*, green-line showed specific genera of *Moist-snuff*, blue-line represent unique genera of *Qiwam* and magenta line specify exclusive genera of *Snus*.

The β–diversity indicates differences in the bacterial community profile between the samples. To calculate β–diversity among the moist STPs, the Bray–Curtis dissimilarity metric was determined from the OTU abundance and exploited in Principal Component Analysis (Goodrich et al., 2014). The Permutational Multivariate Analysis of Variance (PERMANOVA) algorithm on Bray–Curtis dissimilarity was applied to construct Principal Coordinate Analysis (PCoA) plots (Kelly et al., 2015). PERMANOVA analysis of Bray–Curtis dissimilarities revealed that the bacteriome of each group was highly dissimilar (PERMANOVA; *F* = 2.9354, *R*^2^ = 0.49456, *p* < 0.004) (Supplementary Figure 4). The 3D-PCoA plot displayed that the *Moist-snuff* and *Qiwam* samples showed close association to each other and therefore, restrain more related bacteriome profiles (Figure 1A). Next, one sample from *Khaini* (K1) and one sample from *Snus* (S1) was also found to be close to each other and clustered together with *Moist-snuff* and *Qiwam* products and the sample K3 and S2 clustered separately from the other samples (Figure 1A). The interactive PCoA 3D plots at the level of the genus were constructed and dissimilar samples K3 and S2 showed a higher abundance of genera *Actinobacteria* and *Prevotella*, respectively (Figures 1B,C).

Further, a ‘Random Forest’ algorithm was applied to validate the similarity and dissimilarity in bacteriome among the four groups of moist STPs (Figure 1D). The decision tree constructed from the random forest classification recognized distinctive bacteriome in moist STPs. In the error plots identified from random forest analysis, the red line represents overall genera present in moist STPs while distinct genera present in *Khaini*, *Moist-snuff*, *Qiwam*, and *Snus* were indicated by yellow, green, blue, and magenta-line, respectively (Figure 1D). Among all three samples of *Khaini*, one sample contained unique genera and two samples demonstrated overlapping genera with *Qiwam* and *Snus*; whereas *Moist-snuff* and *Qiwam* products showed resemblance with each other. The *Snus* products exhibited a unique genera profile (Figure 1D).

### Taxonomic Distribution of Dominant Bacterial Communities in Moist Smokeless Tobacco Products

**FIGURE 2:**
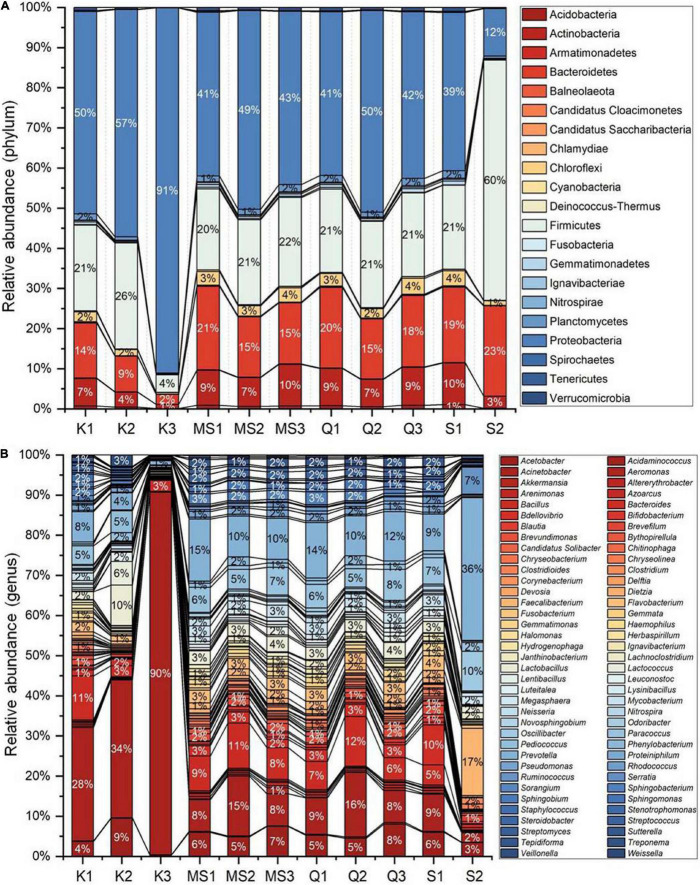
Bacterial phyla of moist smokeless tobacco products. **(A)** The stacked bar showed the relative abundance of bacterial phyla identified in each STPs. The OTUs > 10 reads were represented in their relative abundance. **(B)** The stacked bar showed the relative abundance of bacterial genera identified in each STPs. The OTUs > 100 reads were represented in their relative abundance. Each bacterial phylum is symbolized as a sequential color (red to blue) in the stacked bar graphs with the connecting lines. The entire relative abundance was calculated as 100% for each product.

Bacterial populations recognized in moist STPs were first analyzed at the phyla level (Figure 2A). There were 4 major phyla *Proteobacteria, Firmicutes, Actinobacteria*, and *Bacteroidetes* (range 89–98%) were observed in all moist STPs. The other notable phyla were *Acidobacteria, Chloroflexi, Cyanobacteria, Fusobacteria, Gemmatimonadetes, Spirochaetes*, and *Verrucomicrobia*. The relative abundance of phylum *Proteobacteria* was found to be majorly present in all STPs except S2 (Figure 2A). The highest proportion of *Proteobacteria* was observed in the *Khaini* group (K1–91%, K2–57%, and K3–50%). *Moist-snuff* (MS1–41%, MS2–49%, and MS3–43%), *Qiwam* (Q-41% and Q2–50%), and *Snus* (S1–39%) also showed an increased level of *Proteobacteria* phyla compared to other phyla. In addition, phyla *Firmicutes* was the predominant phyla in S2 (60%) with the lowest presence in K3 (4%) (Figure 2A). The third most prevalent phylum identified in all STPs was *Bacteroidetes* (Figure 2A). However, *Khaini* had a lower abundance (range 2–14%) of *Bacteroidetes* as compared to *Moist-snuff, Qiwam*, and *Snus* (range 15–23%). The phyla *Actinobacteria* was noticeably present in all moist STPs ranging from 1 to 10% (Figure 2A).

A total of 549 genera were identified in all moist STPs and OTUs > 100 reads showed 61 genera were in relative abundance (Figure 2B). The genus *Acinetobacter* was abundantly observed in *Khaini* (90% of K3, 34% of K2, and 28% of K1). Another genus *Prevotella* was found to be significantly high in one of the *Snus* (36% of S2). However, a moderate level of *Prevotella* was observed in *Moist-snuff* (MS1-15%, 10% of MS1 and MS2), *Qiwam* (14% of Q1, 12% of Q3, and 10% of Q2) and *Snus* (9% of S1). The other important genera observed in STPs were *Faecalibacterium* (17% of S2), *Bacillus* (12% of Q2, 11% of K1 and MS2, and 9% of MS1), *Lactobacillus* (10% of K2), and *Ruminococcus* (7% of S2) (Figure 2B).

### Core Bacteriome of Moist Smokeless Tobacco Products

**FIGURE 3:**
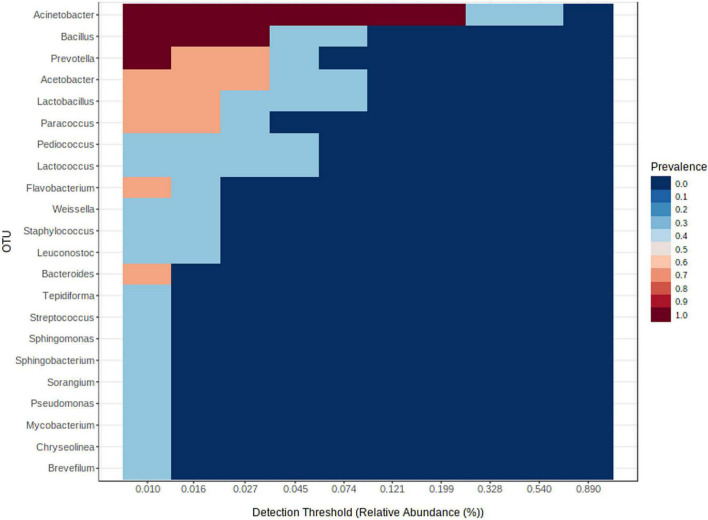
Core bacteriome of moist smokeless tobacco products. The core bacterial genera in moist STPs determined by applying the parameters sample dominance (≥20%) and relative abundance (≥0.2%). Heatmap illustrating the detection threshold and relative abundances of the most dominant bacterial genera in tested moist STPs. The color key shows the range of threshold relative abundance of the individual values.

Despite inter-product variability, there was a core bacteriome identified in the moist STPs that remain unchanged in their composition across different groups of moist STPs. Core bacteriome investigation was executed at the genus level according to sample prevalence ≥ 20% and relative abundance ≥ of 0.2% (Figure 3 and Supplementary Table 4). The 22 core bacterial genera were identified and the prevalence of *Acinetobacter*, *Bacillus*, and *Prevotella* genera was observed in moist STPs (Figure 3). Additionally, *Acetobacter*, *Lactobacillus*, *Paracoccus*, *Flavobacterium*, and *Bacteroides* were found to be the dominant core bacteria in moist STPs (Figure 3).

### Clustering Analysis of Moist Smokeless Tobacco Products and Their Associated Bacterial Genera

The best correlation among samples of moist STPs at the OTU level was determined using the Bray–Curtis index (Supplementary Figure 5). The dendrogram showed similarities between the products and 11 moist STPs clustered into three groups. Group-I consists of two *Khaini* products K1 and K2 whereas K3 was clustered with Q2 and MS2 into a subgroup of Group-II. The high moisture containing products such as *Moist-snuff* and *Qiwam* clustered together into subgroups of Group-II (Supplementary Table 3 and Supplementary Figure 5). Therefore, the high moisture content may lead to similar bacterial diversity in moist STPs. Group-III comprises *Snus* samples S2 and S_2 dup whereas S1 clustered with their respective sample in Group-II which confirmed that the sequencing method produced reproducible data (Supplementary Figure 5).

Additionally, a hierarchical clustering heat map was generated for improved visualization of the distinct bacterial genera abundance across different moist STPs (Supplementary Figure 6). For *Khaini* samples the dominant genera included *Acinetobacter*, *Staphylococcus*, *Panacibacter*, *Citrobacter*, *Pediococcus*, *Lactococcus*, *Weissella*, *Lactobacillus*, and *Leuconostoc*. The *Moist-snuff* samples showed the abundance of genera such as *Mycoplasma*, *Pantoea*, *Filifactor*, *Trueperella*, *Lysinibacillus*, *Marinobacter*, *Frankia*, *Campylobacter*, *Olsenella*, *Aeromonas*, *Dolosigranulum*, *Agromyces*, *Collinsella*, and *Fuerstia* (Supplementary Figure 6). The *Qiwam* products demonstrated the dominance of *Chelativorans*, *Proteus*, *Capnocytophaga*, *Microvirga*, *Methylobacterium*, *Turicibacter*, *Parvibaculum*, and *Simkania* genera. The *Snus* products illustrated the occurrence of genera *Tabrizicola*, *Sulfuritortus*, *Fimbrimonas*, *Anaeromyxobacter*, *Caloramator*, *Immudisolibacter*, *Mannheimia*, *Dermabacter*, *Ruminococcus*, and *Lachnoclostridium* (Supplementary Figure 6). The genera having clinical relevance like *Pseudomonas*, *Haemophilus*, *Actinomyces*, *Neisseria*, *Streptococcus*, *Campylobacter*, *Corynebacterium*, *Porphyromonas*, and *Fusobacterium* were abundant in *Moist-snuff* products while *Prevotella*, *Faecalibacterium* and *Clostridium* were high in *Snus* product S2. The genus *Capnocytophaga* and *Bacillus* was elevated in Q1 and Q2 product, respectively (Supplementary Figure 6).

### Co-occurrence Network of Bacterial Genera Associated With Moist Smokeless Tobacco Products

**FIGURE 4:**
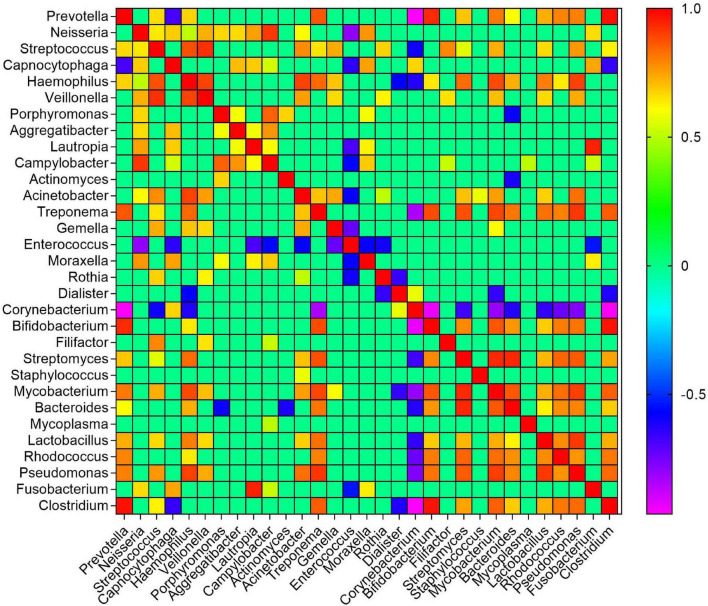
Co-occurrence of bacterial genera in moist smokeless tobacco products. The SparCC correlation of clinically relevant genera was generated and plotted in a heatmap. The scale bar on the right of the plot showed calculated positive and negative correlation values to generate the heatmap. The correlation threshold | >0.3 and *p-*value < 0.05.

To identify potential interactions within the moist STPs bacterial communities, a co-occurrence network at the level of genus was constructed using a compositional robust method SparCC correlation coefficient that formulate a strong assumption of a sparse correlation network (Friedman and Alm, 2012). Overall, the prevalence of 247 genera were found to be considerably different between the groups of moist STPs and to aid interpretation, nodes were colored according to their phylum (Supplementary Figure 7A). Altogether, 245 positive and 238 negative considerable correlations (coefficient correlation > 0.3 and *p*-value < 0.05) were observed between 247 genera (Supplementary Table 5). The co-occurrence pattern of relevant genera, significantly associated with pre-cancer lesion or oral cancer, in moist STPs were examined in detail (Halboub et al., 2020; Sarkar et al., 2021; Srivastava et al., 2021). The genus *Prevotella* was found to be correlated positively with *Clostridium* (SparCC = 0.9719, *p* = 0.0099), *Bifidobacterium* (SparCC = 0.9332, *p* = 0.0099), *Treponema* (SparCC = 0.9332, *p* = 0.0099), *Mycobacterium* (SparCC = 0.8094, *p* = 0.0099), *Rhodococcus* (SparCC = 0.7948, *p* = 0.0099), and *Lactobacillus* (SparCC = 0.7294, *p* = 0.0396) while it was negatively correlated with *Corynebacterium* (SparCC = −0.9622, *p* = 0.0099) and *Capnocytophaga* (SparCC = −0.6792, *p* = 0.0297) (Figure 4 and Supplementary Table 5). *Streptococcus* was positively correlated with *Veillonella* (SparCC = 0.9237, *p* = 0.0099) and *Haemophilus* (SparCC = 0.8739, *p* = 0.0099), whereas *Fusobacterium* showed positive association with *Capnocytophaga* (SparCC = 0.7415, *p* = 0.0297) and *Lautropia* (SparCC = 0.9473, *p* = 0.0099). Another important genera *Pseudomonas* displayed positive concurrence with *Treponema* (SparCC = 0.9149, *p* = 0.0198), *Mycobacterium* (SparCC = 0.9027, *p* = 0.0099), *Haemophilus* (SparCC = 0.8982, *p* = 0.0297), *Prevotella* (SparCC = 0.8000, *p* = 0.0099), *Acinetobacter* (SparCC = 0.8247, *p* = 0.0099), *Bifidobacterium* (SparCC = 0.8186, *p* = 0.0099), *Streptomyces* (SparCC = 0.8592, *p* = 0.0099), and *Clostridium* (SparCC = 0.8345, *p* = 0.0099) (Figure 4 and Supplementary Table 5). The correlation network plot is interactive and the genera *Prevotella* showed the highest abundance in *Snus* products compared to *Khaini*, *Moist-snuff*, and *Qiwam* (Supplementary Figure 7B).

### Biomarker Detection by Linear Discriminant Analysis Effect Size

**FIGURE 5:**
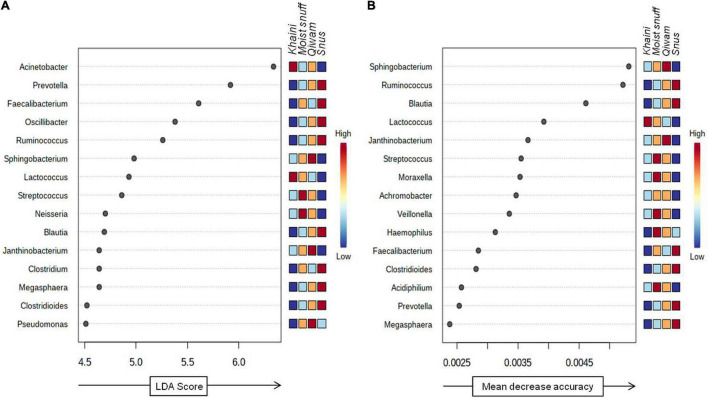
Biomarker analysis of moist smokeless tobacco products-linked bacteriome. **(A)** Linear discriminant analysis Effect Size (LEfSe) of bacteriome present in moist STPs. The significant 15 genera were ranked in declining order as per their LDA scores (*x*-axis). (LEfSe parameters; Taxonomic level-genus, FDR-adjusted *p-*value cut off <0.1, log LDA score > 2.0). **(B)** The significant feature was identified by random forest analysis. The variable importance calculated by mean decrease in accuracy of predictor genera in the Random Forest model and top 15 genera were ranked in increasing order as per their mean decrease accuracy value (*x*-axis). The right heatmap plot designates whether the genera abundance were high (red) or low (blue) in each group of moist smokeless tobacco products.

The robust biomarker of moist STPs was identified using a non-parametric statistical method LEfSe (Segata et al., 2011). LEfSe method discovers features with considerable differential abundance across the moist STPs and established the biomarker bacteriome at the genus level. Fifteen significant taxa were recognized as per the cutoffs values: FDR-adjusted *p*-value < 0.1 and linear discriminant analysis (LDA) > 2.0 (Figure 5A). The LDA score for *Acinetobacter* and *Lactococcus* were highest in *Khaini* products whereas *Streptococcus* and *Neisseria* had high LDA scores in *Moist-snuff* samples (Figure 5A). Further, the LDA score of *Sphingobacterium*, *Janthinobacterium*, and *Pseudomonas* was dominant in *Qiwam*, whilst that of *Prevotella*, *Faecalibacterium*, Oscillibacter, *Ruminococcus*, *Blautia*, *Clostridium*, *Megasphaera*, and *Clostridioides* was highest in *Snus* products (Figure 5A).

Next, to identify bacterial genera that differentiate between phenotypes, a random forest algorithm was applied to bacteriome data (Knights et al., 2011). The significant genera identified with random forest showed a pattern of changes across different groups of moist STPs (Figure 5B). At the genus level, the random forest model brought up *Sphingobacterium, Ruminococcus, Blautia, Lactococcus, Janthinobacterium, Streptococcus, Moraxella, Achromobacter, Veillonella, Haemophilus, Faecalibacterium, Clostridioides, Acidiphilium, Prevotella*, and *Megasphaera* (Figure 5B). The genera *Sphingobacterium, Ruminococcus*, and *Blautia* were the most decisive discriminated genera because of their higher predictive values.

### Functional Capacity of Moist Smokeless Tobacco Products-Linked Bacteriome

The 16s rDNA sequencing data were used to infer the metabolic potential of bacteriome with PICRUSt which is derived from a phylogenetic distance or sequence similarity of identified microbes with the related microorganism whose whole genome has been sequenced and based on Greengenes annotated OTUs (Langille et al., 2013). The MicrobiomeAnalyst utilizes the PICRUSt algorithm and metagenome contributions were computed for all moist STPs based on Kyoto Encyclopedia of Genes and Genomes (KEGG) orthology (KO term) (Kanehisa et al., 2013; Dhariwal et al., 2017). The result containing KO abundance level was generated and in sum 3695 KO terms were observed in the imputed metagenome of moist STPs (Supplementary Table 6). Several KEGG metabolic pathways were identified in moist STPs and their relative abundance in different moist STPs was monitored by MicrobiomeAnalyst (Supplementary Figures 8A,B).

### Nitrogen Metabolism Potential of Moist Smokeless Tobacco-Linked Bacteriome

Microbial reduction of nitrates to nitrite that leads to formation of TSNAs involves the nitrogen metabolism pathway.

**FIGURE 6:**
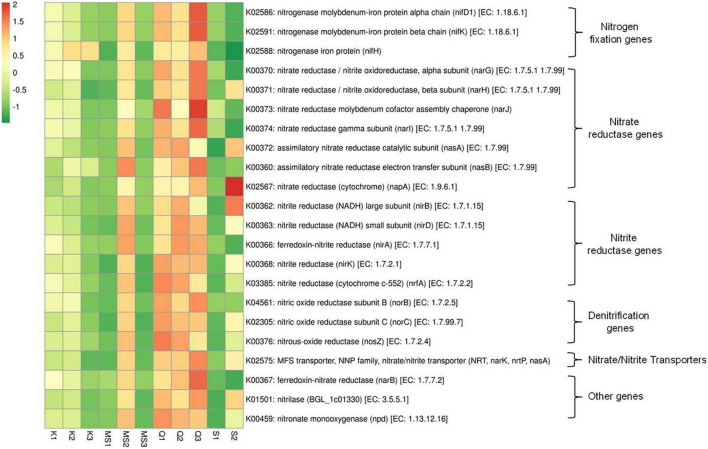
Relative abundance of nitrogen metabolism pathway genes in moist smokeless tobacco products. Heatmap displaying the predicted genes identified using PICRUSt (*y*-axis) based on KEGG database in each sample of moist smokeless tobacco products (*x*-axis). Each column represents a SLT sample and each row nitrogen metabolism gene with relative abundance indicated by color bar.

The two important pathways involved in the extracellular accumulation of nitrite are (i) dissimilatory nitrate reduction *nar* operon that includes regulators (*narXL*), transporters (*narK*) and nitrate reductases (*narZHJI*); (ii) periplasmic nitrate reductase *nap* operon (González et al., 2006). The dissimilatory nitrate reduction pathway genes (*narK*, *narZ*, *narJ*, *narI*, and *narH*) were abundant in the Q3 product and Q1, Q2, and MS2 products showed a significant number of imputed genes of the nitrate reduction pathway (Figure 6 and Supplementary Table 6). The periplasmic nitrate reductases gene *napA* was prevalent in one *Snus* product (S2) and noticeably present in Q3, Q1, and MS2 products. The assimilatory nitrate reductases genes *nasA* and *nasB were* also predicted by PICRUSt and abundantly monitored in *Qiwam* (Q1, Q2, and Q3), *Moist-snuff* (MS2) and *Snus* (S2) products. The predicted genes of nitrite reductases including *nirA*, *nirB*, *nirD*, *nirK*, and *nrfA* were found to be abundant in all three *Qiwam* products and one *Moist-snuff* (MS2) product, whereas *nirB* was also prevalent in one *Snus* product (S2) (Figure 6 and Supplementary Table 6). Another important step in nitrogen metabolism is denitrification in which nitrogenous compounds (nitrate, nitrite, and ammonia) were converted to nitrogen gas (N_2_). All moist STPs contained imputed genes related to denitrification *nosZ* (K00376), *norB* (K04561), and *norC* (K02305) but their abundance was very low (Supplementary Table 4). Further, nitrogen fixation related genes *nifD1* (K02586), *nifH* (K02588), and *nifK* (K02591) were present in all moist STPs and the abundance of *nifD1* and *nifK* were high in Q3, Q1, MS2, Q2, and K1 whereas *nifH* was prevalent in Q3, K2, K3, Q1, MS2, Q2, and K1 (Figure 6 and Supplementary Table 6).

### Antibiotic Drug Resistance Abundance in Bacteriome of Moist Smokeless Tobacco Products

**FIGURE 7:**
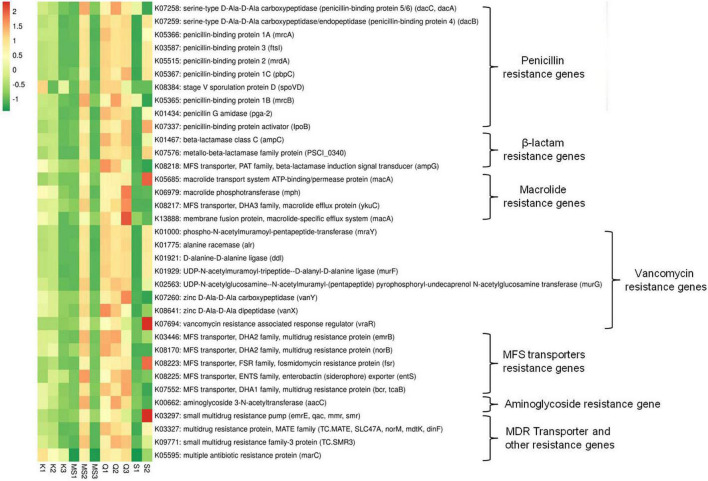
Relative abundance of antibiotics resistance genes in moist smokeless tobacco products. Heatmap displaying the predicted genes identified using PICRUSt (*y*-axis) based on KEGG database in each sample of moist smokeless tobacco products (*x*-axis). Each column represents a SLT sample and each row antibiotic resistance gene with relative abundance indicated by color bar.

The imputed metagenome of moist STPs through PICRUSt assists in the prediction of genes involved in antibiotic drug resistance. The highest number of antibiotic resistance genes was observed in all three products of *Qiwam*, one *Moist-snuff* product (MS2) and one *Snus* product (S2) as compared to *Khaini* products (K1, K2, and K3), MS1, MS3, and S1 products (Figure 7 and Supplementary Table 6). The multiple antibiotic resistance protein *marc* (K05595), penicillin-binding protein 1A *mrcA* (K05366) and penicillin-binding protein-4 (serine-type D-Ala-D-Ala carboxypeptidase/endopeptidase) *dacB* (K07259) was the two most abundant antibiotic resistance genes (Supplementary Table 6). The Q3 product showed genes having higher predicted prevalence such as macrolide-specific efflux system (*macA*, K13888), macrolide phosphotransferase (*mph*, K06979), macrolide efflux protein (*ykuC*, K08217), zinc D-Ala-D-Ala carboxypeptidase (*vanY*, K07260) and multidrug resistance protein (*bcr/tcaB*, K07552) (Figure 7 and Supplementary Table 6). The products Q2 and MS2 showed the prevalence of antibiotic resistance genes like multidrug-resistance protein (*mdtG*, K08161), aminoglycoside 3-*N*-acetyltransferase (aacC, K00662), penicillin-binding protein 1B (*mrcB*, K05365), and penicillin-binding protein 5/6 (*dacC/dacA*, K07258) (Figure 7 and Supplementary Table 6). The sample Q1 displayed the presence of zinc D-Ala-D-Ala dipeptidase (K08641), beta-lactamase induction signal transducer (K082184), and multidrug resistance proteins (*emrB*, K03446 and *norB*, K08170). The S2 products displayed antibiotic resistance genes including vancomycin resistance associated response regulator (*vraR*, K07694), small multi-drug resistance pump (*emrE*, K03297), macrolide transport system ATP binding/permease protein (*macA*, K05685), fosmidomycin resistance protein (*tsr*, K08223), and penicillin-binding protein activator (*lpoB*, K07337) (Figure 7 and Supplementary Table 6).

### Pro-inflammatory and Toxic Effect of Moist Smokeless Tobacco Products-Associated Bacteriome

**FIGURE 8:**
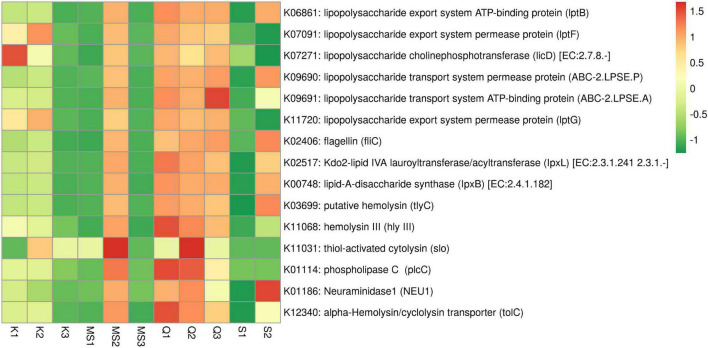
Relative abundance of imputed genes encoding toxins and pro-inflammatory molecules in moist smokeless tobacco products. Heatmap displaying the predicted genes identified using PICRUSt (*y*-axis) based on KEGG database in each sample of moist smokeless tobacco products (*x*-axis). Each column represents a moist STP sample and each row toxin/pro-inflammatory gene with relative abundance indicated by color bar.

Gram-negative bacteria lipopolysaccharide (LPS) is involved in the progression and migration of oral squamous cell carcinoma (OSCC) (He et al., 2015). The LPS inflammatory activity is due to the Lipid-A component that is known to activate pro-inflammatory cytokines (Zhang and Ghosh, 2000; Karpiński, 2019). The ABC transporter complex (*lptBFG*) responsible for LPS transport from the inner to the outer membrane was found in moist STPs and its abundance was high in *Qiwam* and MS2 products. In moist STPs several genes related to LPS biosynthesis were observed (Figure 8 and Supplementary Table 6). The abundance of genes Lipid-*A*-disaccharide synthase (*lpxB*) and Kdo2-lipid IVA lauroyltransferase/acyltransferase (*lpxL*) which were involved in LPS synthesis significantly increased in *Qiwam* (Q1, Q2, and Q3), MS2 and S2 products (Figure 8 and Supplementary Table 6).

Another potent pro-inflammatory molecule is Flagellin which participates in the motility of bacteria. Flagellin coding gene *fliC was* found in all moist STPs and their abundance was high in S2, Q3, Q2, MS2, and Q1 as compared to K2, K1, S1, MS3, K3, and MS1 (Figure 8 and Supplementary Table 6). However, the genes involved in the synthesis of other pro-inflammatory molecules like peptidoglycan, teichoic acid and lipoteichoic acid (K03739 and K03740) were not observed in any moist STPs (Supplementary Table 6).

Further, an important category of genes is related to bacterial toxins because these toxins can participate in the pathogenesis of inflammation leading to several diseases (toxinoses) and can induce genomic damage that can lead to neoplastic transformation of epithelial cells (La Rosa et al., 2020). The STP-associated bacterial toxins identification based on KEGG orthology displayed that bacterial species having toxins genes were present in moist STPs. The highest sequence hits were observed for alpha-Hemolysin/cyclolysin transporter gene *tolC* (K12340), followed by putative hemolysin gene (*tlyC*, K03699), phospholipase-C gene (*plcC*, K01114), Neuraminidase1 gene (*NEU1*, K01186), hemolysin III gene (hly III, K11068) and thiol-activated cytolysin gene (*slo*, K11031) (Figure 8 and Supplementary Table 6). The moist STPs Q1, Q2, MS2, and S2 showed a high abundance of toxin genes whereas Q3, K1, and K2 also contain a significant prevalence of these genes compared to K3, MS1, MS3, and S1 products (Figure 8 and Supplementary Table 6).

### Taxon Set Enrichment Analysis of Moist Smokeless Tobacco Product Bacteriome

Next, we performed a Taxon Set Enrichment Analysis (TSEA) of all significant genera using the MicrobiomeAnalyst tool and examined them across 239 known taxon set linked with host-intrinsic factors such as age and diseases (Chong et al., 2020). The bacteriome of moist STPs showed strong correlations with colorectal cancer, Crohn’s disease and Rheumatoid arthritis (Table 2). Further, we parsed the bacteriome of moist STPs with 53 taxon sets associated with microbiome-intrinsic factors such as microbe motility and shape. The bacteriome of moist STPs displayed a significant correlation with indole producers and mucin degraders (Table 3).

**TABLE 2:** Correlation of host-intrinsic factors (diseases) with bacteriome of moist smokeless tobacco products.

S. no.	Host-intrinsic factors	Total	Expected	Hits	Raw *p*	Holm *p*	FDR
(1)	Colorectal carcinogenesis (increase)	7	0.883	6	0.0000236	0.00561	0.00561
(2)	Crohn’s disease (increase)	21	2.65	10	0.0000817	0.0194	0.00973
(3)	Melatonin	3	0.379	3	0.00198	0.468	0.118
(4)	Bristol av.stool.freq	25	3.15	9	0.00224	0.526	0.118
(5)	Rheumatoid arthritis (China, increase)	30	3.79	10	0.00248	0.58	0.118

**TABLE 3:** Correlation of microbiome-intrinsic factor with bacteriome of moist smokeless tobacco products.

S. no.	Microbiome-intrinsic factors	Total	Expected	Hits	Raw *p*	Holm *p*	FDR
(1)	Indole producers	83	8.63	26	0.0000000661	0.00000344	0.00000344
(2)	Mucin degraders	11	1.14	7	0.0000272	0.00139	0.000707
(3)	Folate consumer	1	0.104	1	0.104	1	1
(4)	Riboflavin producers	5	0.52	1	0.423	1	1
(5)	Butyrate producers	14	1.46	2	0.436	1	1

## Discussion

The bacteriome of moist STPs was established by high throughput sequencing of 16S rDNA (V3-V4 region) sequence present in genomic DNA isolated from the STPs and first time expansively analyzed (community profiling, comparative analysis, and functional prediction) by MicrobiomeAnalyst tool (Dhariwal et al., 2017). Several previous studies have identified culturable and non-culturable microorganisms in STPs (Rubinstein and Pedersen, 2002; Han et al., 2016; Onuorah Samuel, 2016; Tyx et al., 2016, 2020). The genera such as *Bacillus*, *Lactobacillus, Staphylococcus, Corynebacterium, Streptococcus, Prevotella, Rothia, Pantoea, Veillonella, Propionibacterium, Fusobacterium, Actinomyces, Lactobacillus, Sphingomonas, Marinilactibacillus, Oceanobacillus*, and *Porphyromonas* were recognized in different STPs (Rivera and Tyx, 2021; Sajid et al., 2021).

In our study, we focused on the moist STPs because high moisture content plays a significant role in the increased level of TSNAs by facilitating the growth of the microorganisms (Gholizadeh et al., 2016; Han et al., 2016). An *in vitro* assay using epithelial cells (AMOL-III) from the oral leukoplakia of a *Khaini* user and treatment with aqueous extract of *Khaini* showed alteration in the expression of proteins involved in cell cycle regulation and DNA methylation, suggesting the role of *Khaini* in oral carcinogenesis (Rohatgi et al., 2005). Other moist STPs category consumed as *Snus* contains high levels of carcinogenic TSNAs (23.1–61.2 μg/g) (Stepanov et al., 2015). *Qiwam* products also retain a significant level of TSNAs (5.43–22.2 mg/kg) (Tricker and Preussmann, 1989).

The α–diversity of bacteriome present in different moist STPs was determined and we have observed that the species richness (α-diversity) was more in STPs having high moisture content such as *Moist-snuff* and *Qiwam* compared to products having low moisture levels like *Khaini* and *Snus*. In contrast, Tyx et al. (2016) observed that American *Dry snuff* products exhibited elevated overall species diversity compared to *Moist-snuff* samples. Al-Hebshi et al. (2017) found the highest species richness in Swedish *Snus* products and lowest for the Yemeni *Shammah* product. Likewise, Monika et al. (2020) observed that Indian *Snus* products have increased α–diversity as compared to less moist STPs like *Chewable tobacco* and *Snuff*. The prevalence of bacteria in moist STPs may be due to the hot and high humid ambiance of India which can facilitate the growth of microorganisms involved in increasing the fermentation rate of alkaloids present in STPs to carcinogenic TSNAs. Further, β–diversity analysis of moist STPs, based on Bray–Curtis dissimilarity metric, revealed that the products of *Moist-snuff* (MS1, MS2, and MS3) and *Qiwam* (Q1, Q2, and Q3) clustered together and a clear bacterial community similarity was localized between *Moist-snuff* and *Qiwam* products whereas distinct separation of *Khaini* and *Snus* products was observed. Following our study, American *Moist-snuff* products also clustered mutually after PCoA analysis based on Weighted UniFrac distances (Tyx et al., 2016; Al-Hebshi et al., 2017).

The moist STPs contain complex communities of bacterial species. The dominant phylum in all moist STPs was *Proteobacteria* followed by *Firmicutes*, the findings being similar to a previous study (Zhou et al., 2020). However, studies conducted on American moist STPs reported *Firmicutes* as the most abundant phylum (Han et al., 2016; Tyx et al., 2016; Al-Hebshi et al., 2017). In our study, we targeted the V_3_-V_4_ section of the 16S rRNA gene and observed the several abundant bacterial genera *Acetobacter*, *Acinetobacter*, *Bacillus*, *Bacteroides*, *Faecalibacterium*, *Lactobacillus*, *Oscillibacter*, *Paracoccus*, *Prevotella*, *Pseudomonas*, and *Ruminococcus*. However, a study on American *Moist-snuff* products metagenomic analysis (based on the V_4_ region of the 16S gene alone) observed several predominant genera *Tetragenococcus*, *Aerococcus*, *Alloiococcus*, and *Staphylococcus* (Tyx et al., 2016). While, another metagenomic study on American Moist-snuff products using the V_1_–V_3_ segment of the 16S rRNA gene identified genera *Paenibacillus*, *Oceanobacillus*, and *Bacillus* (Al-Hebshi et al., 2017). Further, a study on Indian STPs (Chewable tobacco, Snus, and Snuff) using entire 16S gene sequencing and analysis established the abundance of genera *Staphylococcus*, *Bacillus*, *Corynebacterium*, *Virgibacillus*, *Brevibacterium*, *Rothia*, *Veillonella*, and *Fusobacterium* (Monika et al., 2020).

The genera co-occurrence network analysis identified several significant relationships within the bacteriome of moist STPs. As revealed in our correlation analysis, the genera involved in the development of oral diseases showed a positive correlation independent of their phyla. Compared with previous studies on bacteriome of STPs, our study, for the first time identified numbers of distinguishing genera using the LEfSe method and random forest analysis. We have identified 15 genera associated directly with the moist STPs.

The most important pathway involved in TSNAs formation is the nitrogen metabolism pathway. Microbial fermentation forms nitrite from nitrate which reacts with numerous tobacco alkaloids to form different carcinogenic TSNAs molecules (Wang et al., 2017). During microbial respiration under anoxic conditions, nitrate reduction is an alternative respiratory pathway, nitrate acts as a terminal electron acceptor in place of oxygen, contributing to the oxidation of NADH (Igamberdiev and Hill, 2004). The nitrate is converted to nitrite by cytosolic nitrate reductase (*nas*, *nap*, and *nar*) and by membrane-bound nitrate reductase (Igamberdiev and Hill, 2004). Expression of nitrate reductases was found during the hypoxic condition that may result in extracellular nitrite accumulation during aging or storage of tobacco/tobacco products (Nishimura et al., 2007). Further, several bacteria contain nitrite exporting enzymes to regulate the level of nitrite as it can be toxic to the microbial cell. The nitrate and nitrite transport process are carried out by nitrate/nitrite anti-porters or nitrite extrusion transporters and determine the extracellular nitrite levels (Alvarez et al., 2019). Excreted nitrite can be metabolize by microbes having assimilatory or dissimilatory (denitrifying) pathways (Averill, 1996; Luque-Almagro et al., 2011). Furthermore, under optimal conditions *N*-nitrosation reaction of alkaloids with nitrite results in the formation of TSNAs (Wang et al., 2017). A few studies identified nitrogen metabolism genes in American STPs and Sudanese Toombak by whole metagenome and 16S rRNA gene metagenomics (Tyx et al., 2016; Rivera et al., 2020). Tyx et al. (2016) observed that the nitrate reductase genes (*narGHJI*), nitrite reductase genes (*nirABC*) and nitrate/nitrite transporters genes were significantly abundant in American dry snuff products. In our study, we have observed that *narGHJI* and *nirABDK* were abundant in moist STPs like *Qiwam* and *Moist-snuff*. The enhanced level of TSNAs in *Qiwam* may be due to a high level of the nitrogen metabolizing enzymes being able to contribute to the synthesis of TSNAs. Therefore, the identification of bacterial species performing nitrogen metabolism is crucial to decipher the carcinogenic potential of bacteria present in moist STPs.

A serious global threat of antimicrobial resistance spread lead to the emergence of multidrug-resistance bacteria or “Superbug” (Aslam et al., 2018). The spread of antibiotic resistance genes to oral microbiota of SLT users can be attributed to the practice of smokeless tobacco consumption (Lacoma et al., 2019). The whole metagenome sequencing and analysis of American-STPs showed the presence of several antibiotic resistance genes associated with resistance to β–lactam, penicillin, vancomycin, macrolides, aminoglycosides antibiotics, and other genes encode for multidrug transporters and efflux pumps (Rivera et al., 2020). The imputed metagenome of Indian-moist STPs like Q1, Q2, Q3, MS2, and S2 displayed a prevalence of antibiotic resistance genes having the potential to deactivate antibiotics. The multidrug efflux pumps of the major facilitator superfamily (MFS) can uniport small molecules and provide a noteworthy mechanism of bacterial resistance to antimicrobial compounds (Kumar et al., 2016). Several MFS transporters resistance genes were identified in moist STPs. Hence, moist STPs used in India can be a source of antibiotic resistance genes and are capable of spreading these genes to human microbiota and make them difficult to treat.

Bacterial association with mucosal lining can deliver bacterial products, like LPS (Gram-negative bacteria) that stimulate many cell types and can contribute to OSCC progression (Kurago et al., 2008). LPS can induce cytokines discharge (IL-6, IL-1β, IL-8, and TNF-α) upon attachment with the toll-like receptor (TLR receptor) causing the LPS induced inflammation (Zhang and Ghosh, 2000; Karpiński, 2019). Additionally, LPS activate TLR-4 of cancer cells and assist tumor cells immune-escape by preventing the action of cytotoxic T cells or natural killer (NK) cells (Huang et al., 2005). Gram-negative bacterium *Shigella flexneri* can inhibit apoptosis by suppressing the effector caspase activity by direct attachment of lipopolysaccharide (LPS) with caspases (Günther et al., 2020). The predicted metagenome of moist STPs include several genes related to LPS transport (ABC transporter complex, *lptBFG*) and their dominance was elevated in *Qiwam* and *Moist-snuff*. The abundance of LPS in moist STPs can provide cancer supporting environment and help in the progression of oral cancer in SLT users.

Studies are suggesting that the microbial-derived toxins (endotoxins, exotoxins and mycotoxins) may contribute to the health risks of STPs (Pauly and Paszkiewicz, 2011; Han et al., 2016). Gram-positive bacterial genera were found to produce hemolysins (pore-forming toxins). An extensively studied Pneumolysin (thio-activated cytolysin, K11031) secreted by *Streptococcus pneumonia* can induce cell death and inflammation by pore-forming cytolytic activity or by provoking the necrosis and program cell death pathways (Nishimoto et al., 2020). We observed that the abundance of Pneumolysin was high in Q2 and MS2 products. Therefore, the presence of Pneumolysin in these products can be attributed to generating host tissue injury especially oral layer damage during chewing of STPs.

In this study bacteriome of moist STPs had a strong correlation with increased colorectal cancer in humans. This observation is corroborated with previous observation where *Fusobacterium nucleatum* (a Gram-negative bacterium) was found to be associated with colorectal cancer and oral cancer (Shang and Liu, 2018; Fujiwara et al., 2020). As we observe that the *Fusarium* genus is abundant in the *Moist-snuff* products, therefore, it can be postulated that the presence of *Fusarium* sp. in moist STPs products can contribute to the development of colorectal and oral cancer. Further, the mucous layer provides protection to oral cavity against pathogens. Initiation of pathogenesis is linked with mucin degradation by the bacteria because it would damage the protective host mucosal surfaces (Derrien et al., 2010). We have observed that TSEA analysis reveal the presence of mucin-degrading bacteria in moist STPs. Hence, mucin degradation in the oral cavity of SLT users by mucin-degrading bacteria can contribute to oral carcinogenesis.

Further, in this study, we collected the STPs from a restricted geographical location due to Covid-19 pandemic travel constraints. Since microbial content of STPs is dependent upon the climatic and storage conditions which may vary across the country. Therefore, a broad spectrum of STPs from different regions of India needs to be inspected for a more definitive outcome.

## Conclusion

All moist STPs harbor diverse bacteriome with the prevalence of harmful bacteria genera *Acinetobacter*, *Bacillus*, *Prevotella*, *Faecalibacterium*, and *Pseudomonas*. *Moist-snuff*, and *Qiwam* products were more significantly diverse and showed similar bacterial diversity than *Khaini* and *Snus* products irrespective of brand, type and manufacturer of the products. The core bacteriome was present in most or all moist STPs tested and showed an abundance of genera *Acinetobacter*, *Bacillus*, *Prevotella*, Acetobacter, *Lactobacillus*, *Paracoccus*, *Flavobacterium*, and *Bacteroides*. The STP-associated bacteriome has significant metabolic potential to contribute to TSNAs synthesis by nitrogen metabolism. The presence of antibiotic drug resistance microbes in the STPs can passively transfer resistance to the oral microbiota of SLT users and may contribute to oral cancer. Moreover, delivery of several bacterial pro-inflammatory components and toxins molecules to the host during STP intake can contribute to the development of oral cancer. Hereafter, identification of carcinogenic potential of bacterial population and their products will provide a detailed insight into oral cancer induction in SLT users and provide a basis to regulate the use of STPs.

## Data Availability Statement

The datasets presented in this study can be found in online repositories. The names of the repository/repositories and accession number(s) can be found below: https://www.ncbi.nlm.nih.gov/sra; PRJNA767533.

## Author Contributions

MS: experiments, data curation, data analysis, literature review, and writing original draft. SS: literature review and manuscript preparation. AmK: data validation and review and editing. AnK and HS: review and editing. MB: conceptualization, supervision, investigation, project administration, funding acquisition, data interpretation, validation, review, and editing. All authors contributed to the article and approved the submitted version.

## Conflict of Interest

The authors declare that the research was conducted in the absence of any commercial or financial relationships that could be construed as a potential conflict of interest.

## Publisher’s Note

All claims expressed in this article are solely those of the authors and do not necessarily represent those of their affiliated organizations, or those of the publisher, the editors and the reviewers. Any product that may be evaluated in this article, or claim that may be made by its manufacturer, is not guaranteed or endorsed by the publisher.

## Footnotes

1
https://www.bioinformatics.babraham.ac.uk/projects/trim_galore/

2
https://www.microbiomeanalyst.ca/

## Supplementary Material

Supplementary Table 1 Barcode sequences.

Supplementary Table 2 OTUs table.

Supplementary Table 3 Characteristics of moist smokeless tobacco products.

Supplementary Table 4 Core microbiome values.

Supplementary Table 5 Correlation values and correlation matrix.

Supplementary Table 6 KEGG orthology (KO) terms.
