# Supplementary material for: Bacteriome of Moist Smokeless Tobacco Products Consumed in India With Emphasis on the Predictive Functional Potential
Source: Front Microbiol. 2021 Dec 24;12:784841. doi: 10.3389/fmicb.2021.784841 (PMC8740325; doi:10.3389/fmicb.2021.784841)
Supplement: Supplementary Table 3 — Characteristics of moist smokeless tobacco products. [file Table_3.docx]

**S3 Table.** Characteristics of moist smokeless tobacco products

| **S. No** | **Category** | **Product ID** | **No. of raw reads (in millions)** | **GC Content** | **Moisture content** | **pH** |
| --- | --- | --- | --- | --- | --- | --- |
| 1. | *Khaini* | K1 | 0.4 M | 55% | 16.005 | 6.46 |
|  |  | K2 | 0.4 M | 54% | 17.005 | 6.77 |
|  |  | K3 | 0.4 M | 52.5% | 14.208 | 5.88 |
| 2. | *Moist snuff* | MS1 | 0.2 M | 55% | 50.190 | 10.01 |
|  |  | MS2 | 0.4 M | 55% | 53.303 | 10.19 |
|  |  | MS3 | 0.2 M | 55.5% | ND | 9.63 |
| 3. | *Qiwam* | Q1 | 0.4 M | 55% | ND | 5.65 |
|  |  | Q2 | 0.4 M | 55% | ND | 5.58 |
|  |  | Q3 | 0.6 M | 54.5% | ND | 5.37 |
| 4. | *Snus* | S1 | 0.2 M | 55% | 22.410 | 10.29 |
|  |  | S2 | 0.2 M | 54% | 18.305 | 10.25 |

ND = not determined due to presence of additives. The physical appearance showed high moisture level in MS3, Q1, Q2, and Q3. (pH and moisture data not published)
