# Supplementary figures and images for: Bacteriome of Moist Smokeless Tobacco Products Consumed in India With Emphasis on the Predictive Functional Potential
Source: Front Microbiol. 2021 Dec 24;12:784841. doi: 10.3389/fmicb.2021.784841 (PMC8740325; doi:10.3389/fmicb.2021.784841)

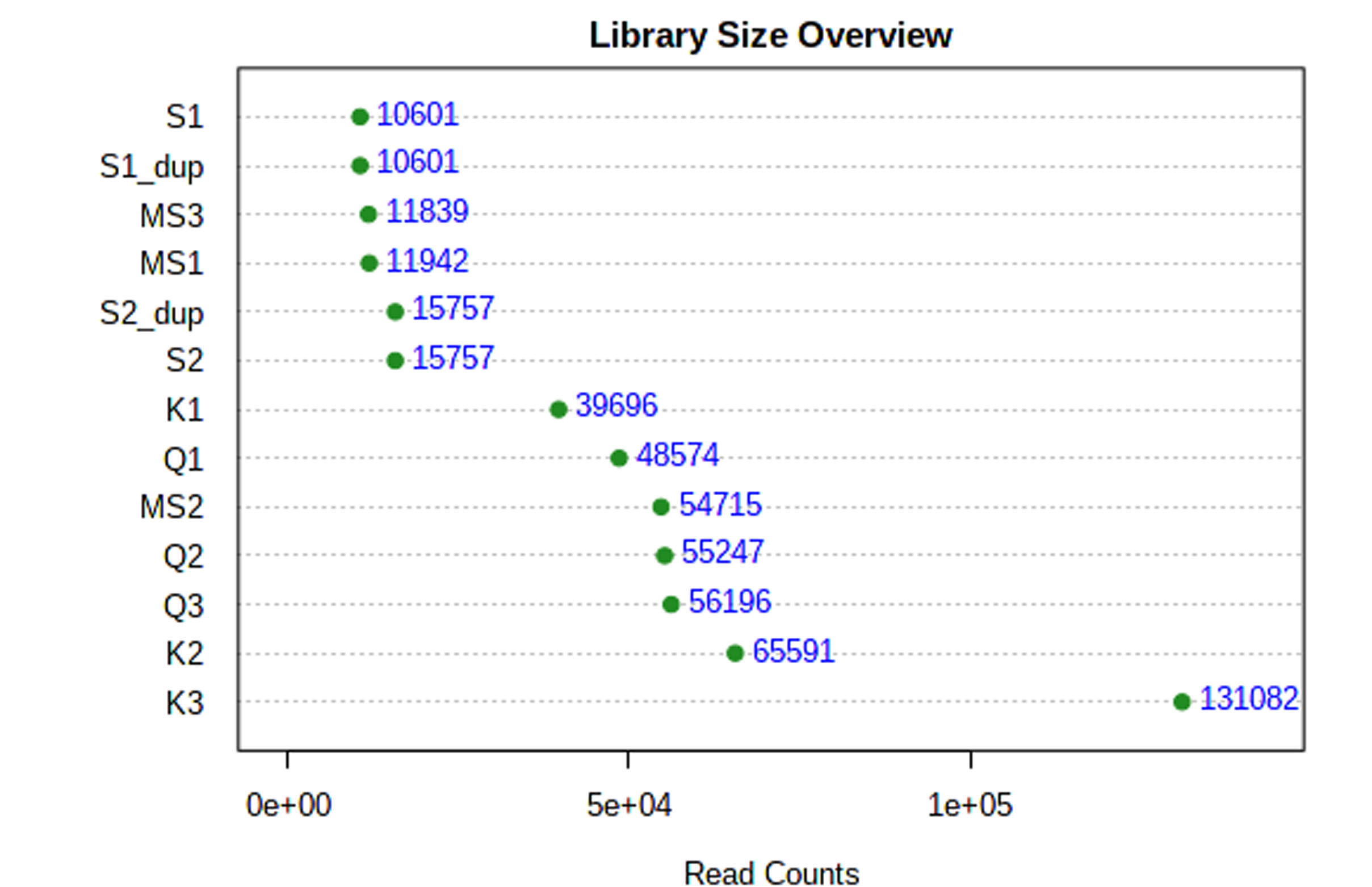

Supplement: Supplementary file 7 [file Image_1.JPEG]

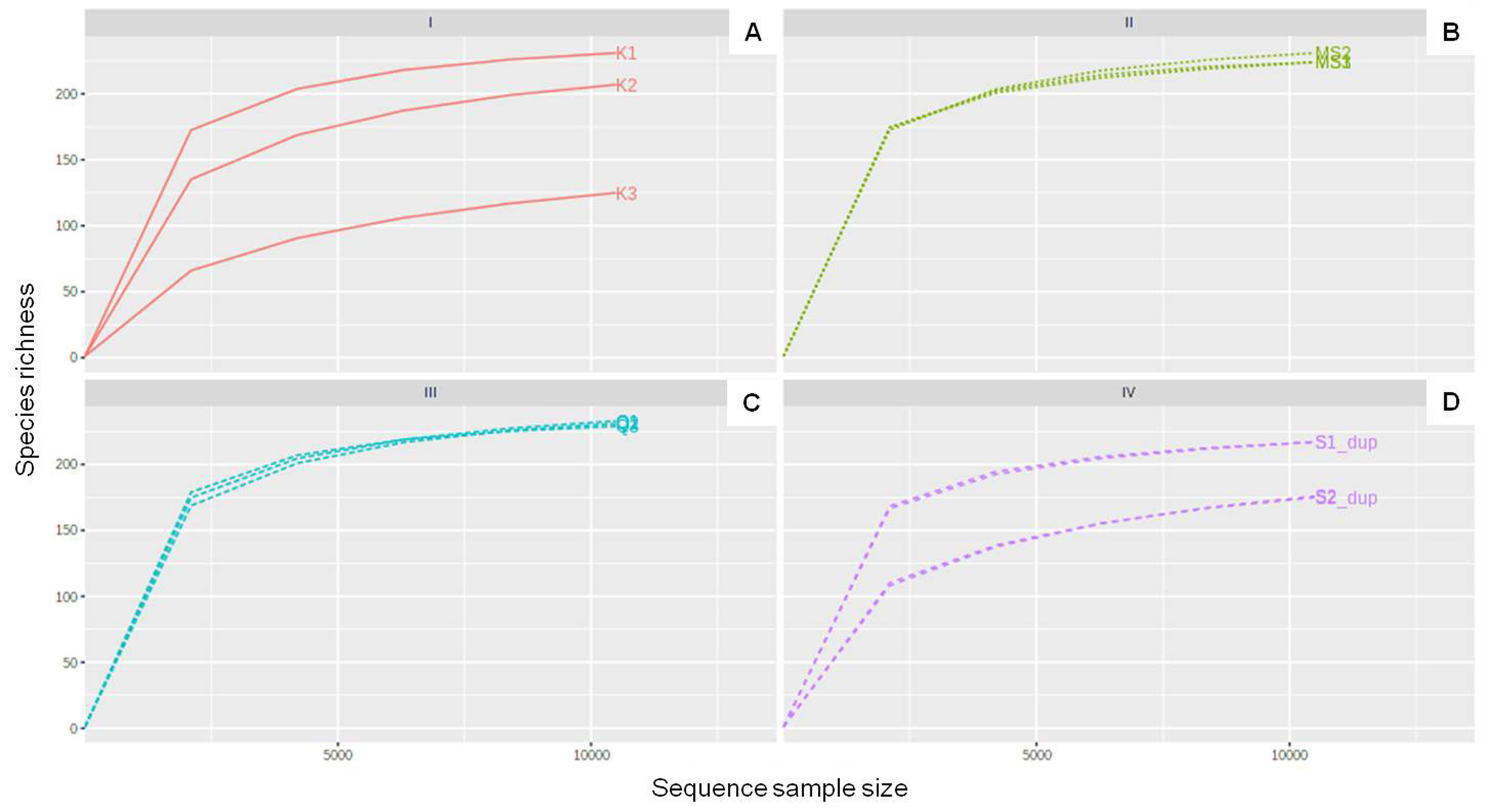

Supplement: Supplementary file 8 [file Image_2.JPEG]

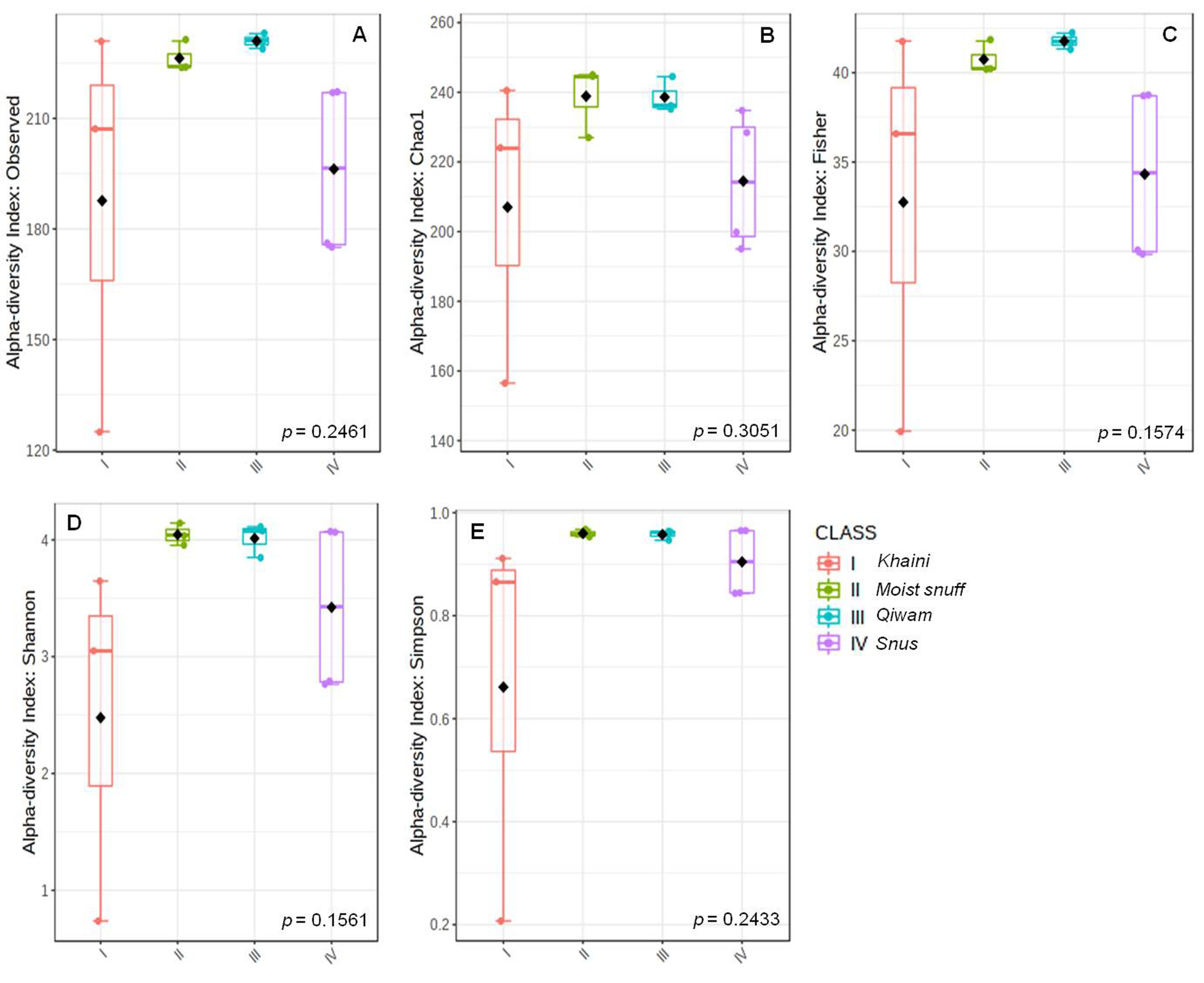

Supplement: Supplementary file 9 [file Image_3.JPEG]

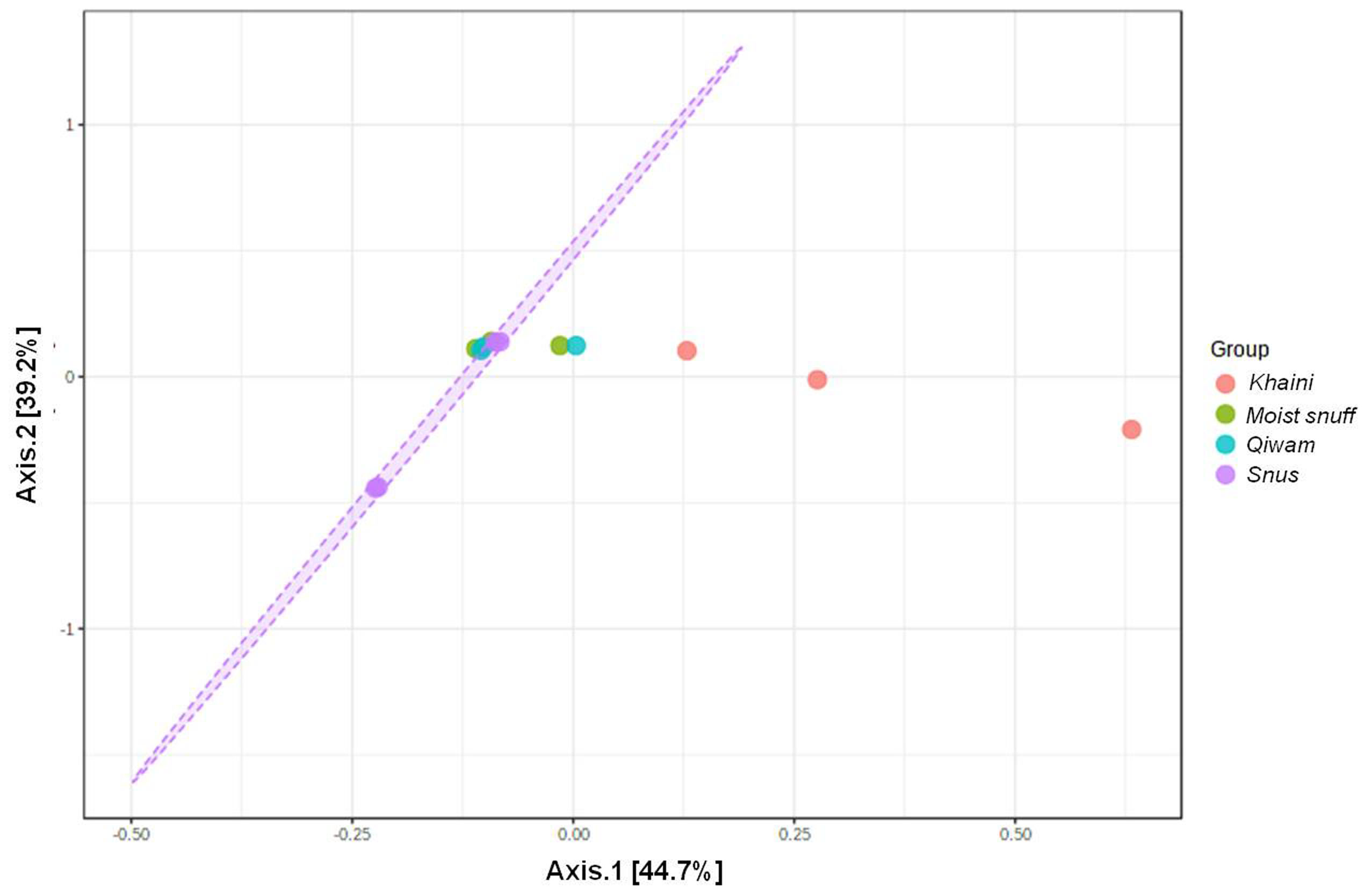

Supplement: Supplementary file 10 [file Image_4.JPEG]

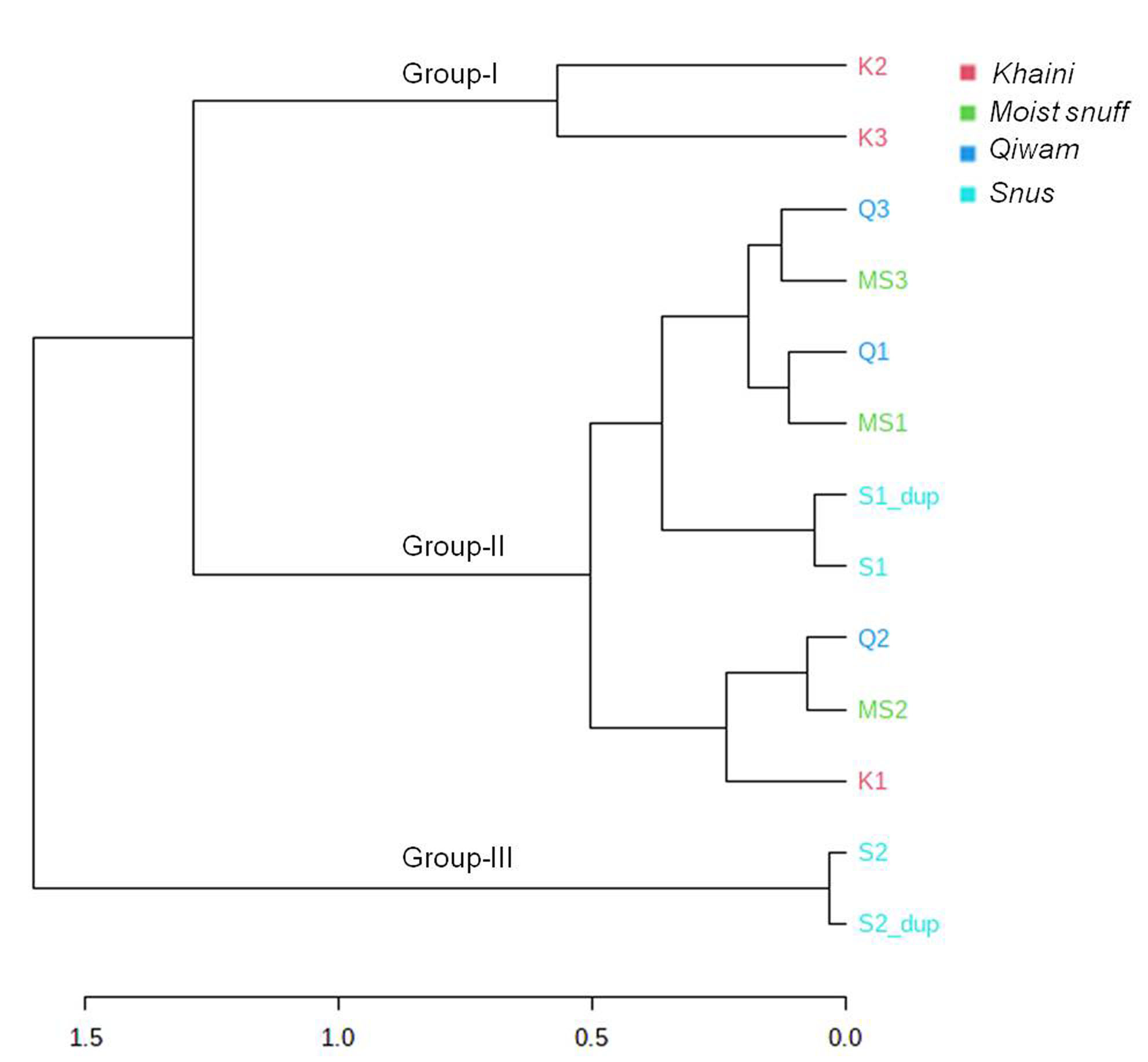

Supplement: Supplementary file 11 [file Image_5.JPEG]

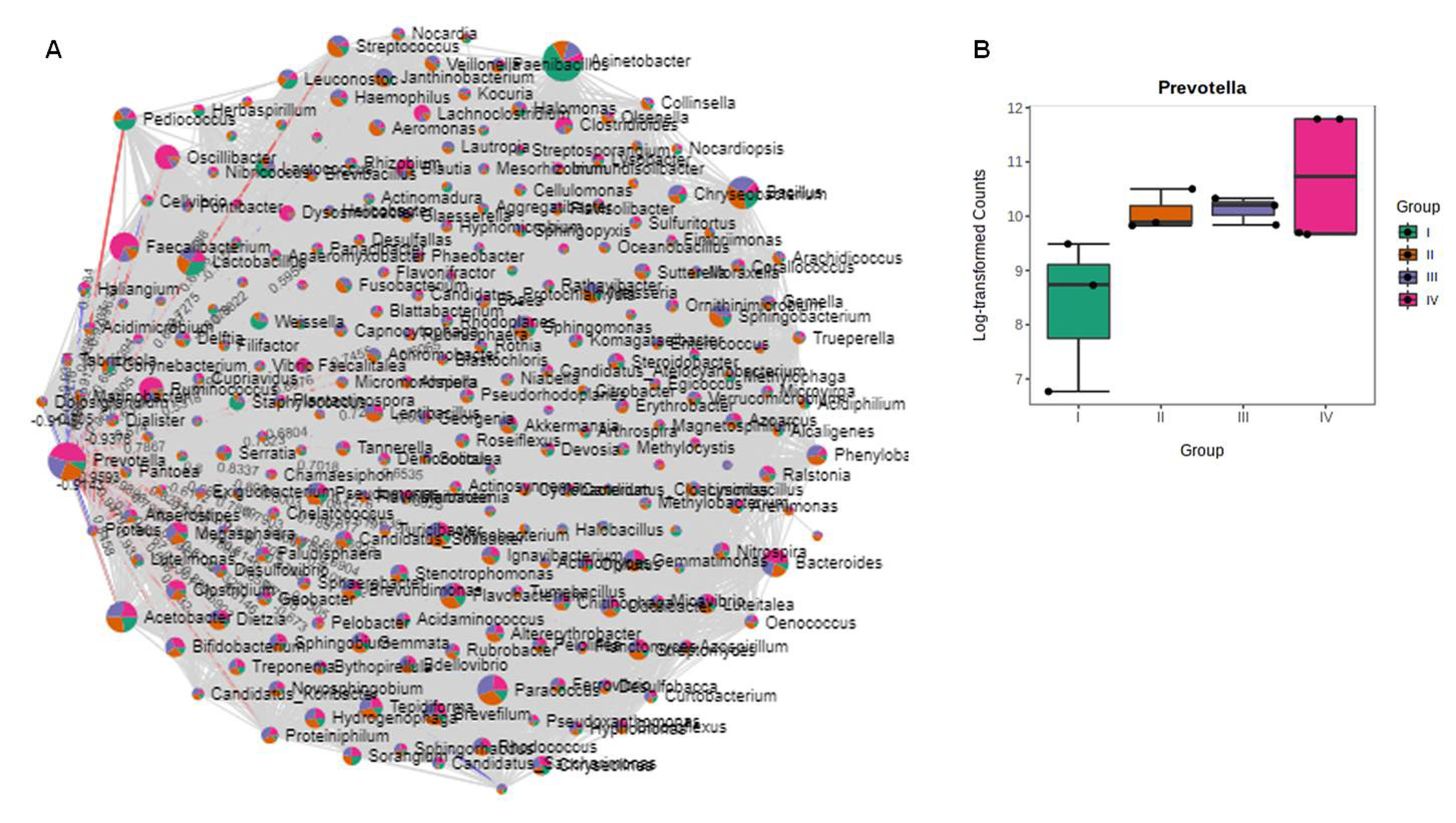

Supplement: Supplementary file 13 [file Image_7.JPEG]

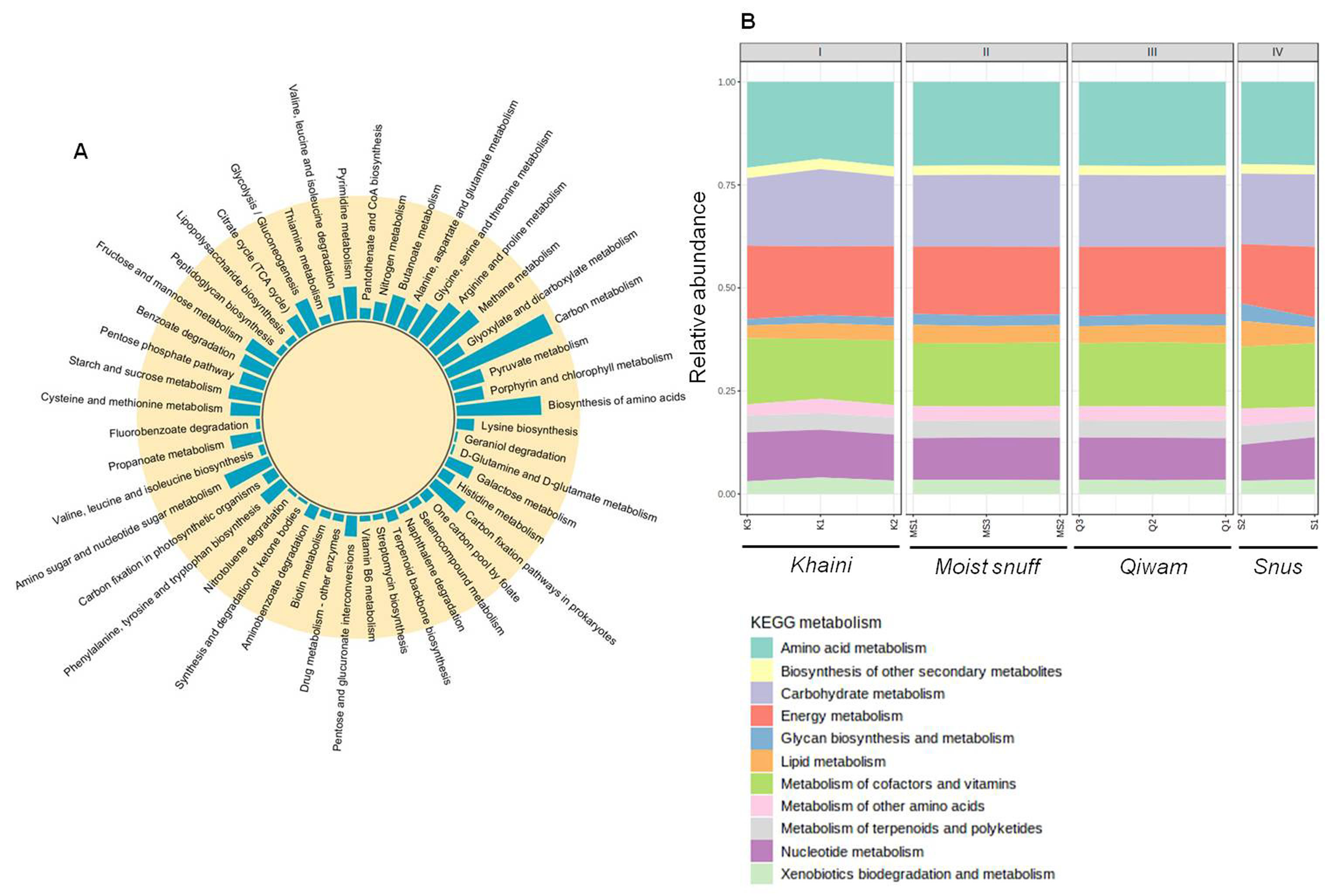

Supplement: Supplementary file 14 [file Image_8.JPEG]
